# Atypical Presentation: Metastatic Uveal Melanoma in a Young Patient without Visual Complaints

**DOI:** 10.3389/fonc.2017.00099

**Published:** 2017-05-18

**Authors:** Pedro Grachinski Buiar, Sérgio Jobim de Azevedo

**Affiliations:** ^1^Department of Medical Oncology, Hospital de Clínicas de Porto Alegre, Porto Alegre, Brazil

**Keywords:** uveal, melanoma, metastasis, young, atypical

## Abstract

**Background:**

Uveal melanoma is a rare and aggressive subtype of melanoma, with singular characteristics that separate it from the most famous cutaneous melanoma. This uncommon condition becomes even rarer if we look at young population. Common chemotherapy regimens does not work with this aggressive disease in its metastatic scenario, and the new armament like targeted and immunotherapies are still looking for more robust evidence.

**Case presentation:**

We report a rare case of uveal melanoma in a patient younger than 20 years, with abdominal pain as his initial complaint. He did not present the typical visual symptoms of the primary site because of an auto accident suffered 4 months before the presentation, letting him blind of the eye affected by the tumor development.

**Conclusion:**

There is always a possibility of the diagnosis of uveal melanoma in cases with associated isolated hepatic metastases, even in a young population, where this hypothesis is often rejected by the epidemiological frequency of other tumors. This rare case is a useful example.

## Introduction—Case Presentation

A healthy 17-year-old male Caucasian presented to emergency department because of vomiting (1–2 episodes per day, not preceded by nausea) associated with epigastric pain and feeling of gastric fullness for 2 weeks. He also reported sudden weight loss of 3.5 kg during this period, noted after onset of vomiting. He had an normal appetite, no fever, or no other concomitant symptoms. Intact Bowel and urinary habits. He was on no chronic medications or drugs and had not yet started sexual activity. He lived on the rural zone, with adequate sanitary conditions, working with farm animals (pigs and chickens) since childhood and exposing himself to solar radiation daily without regular protection. He had no contact with his biological mother (for unknown reasons). His immunization schedule was unknown too. There was no significant disease history in his biological family. As a personal history, he had cranial trauma due to car accident approximately 4 months before the presentation, resulting in multiple facial skin lacerations, associated with orbital fracture, right cornea, and anterior chamber of right eye injuries. This trauma resulted in total vision loss in the right eye. The radiological images taken in the emergency hospital during this event were lost by the patient and family.

At physical examination, the patient was pale, non-cyanotic, non-icteric, and eupneic. Scars all over the face and conjunctival hyperemia were associated with asymmetric pupillary deformation of the blinded right eye. There were no peripheral lymphadenopathy and no increase or change in thyroid level. Cardiopulmonary examination was normal. Abdominal distention due to massive hepatomegaly, extending through almost the entire abdominal cavity, with irregular nodulated surface was found. Palpable spleen 4 cm below left costal margin was seen. Testicles were normal to the examination. His skin was evaluated by a dermatologist team, without suspected injuries among a great deal of nevi.

Abdomen ultrasonography confirmed hepatomegaly with diffusely heterogeneous echogenicity at the expense of innumerable low echogenic nodules, compromising the hepatic parenchyma diffusely, reaching up to 7.1 cm. Ascitic fluid was present in a small amount.

### Laboratory Tests

The laboratory test results were as follows: hemoglobin, 13.1 mg/dL; leukocyte count, 4,960 cells/μL (normal series); platelets, 171 × 10^3^/μL; prothrombin time—INR, 1.3 (14.2 s); activated partial thromboplastin time, 38 s; urea, 32 mg/dL; creatinine, 1,12 mg/dL; alanine aminotransferase, 67 IU/L; aspartate aminotransferase, 62 IU/L; sodium, 138 mEq/L; potassium, 4.5 mEq/L; lipase, 19 U/L; glucose, 125 g/dL; albumin, 3.2 g/dL; alkaline phosphatase, 426 IU/L; gama-glutamil transferase, 324 IU/L; lactate dehydrogenase, 1,620 IU/L; total bilirubin, 0.32 mg/dL; alpha-fetoprotein, 0.86 ng/mL; human chorionic gonadotropin, 0,8 mlU/mL; and negative serologies for Chagas disease, hepatitis, human immunodeficiency virus, cytomegalovirus, Epstein–Barr virus, and toxoplasmosis.

**Figure 1:**
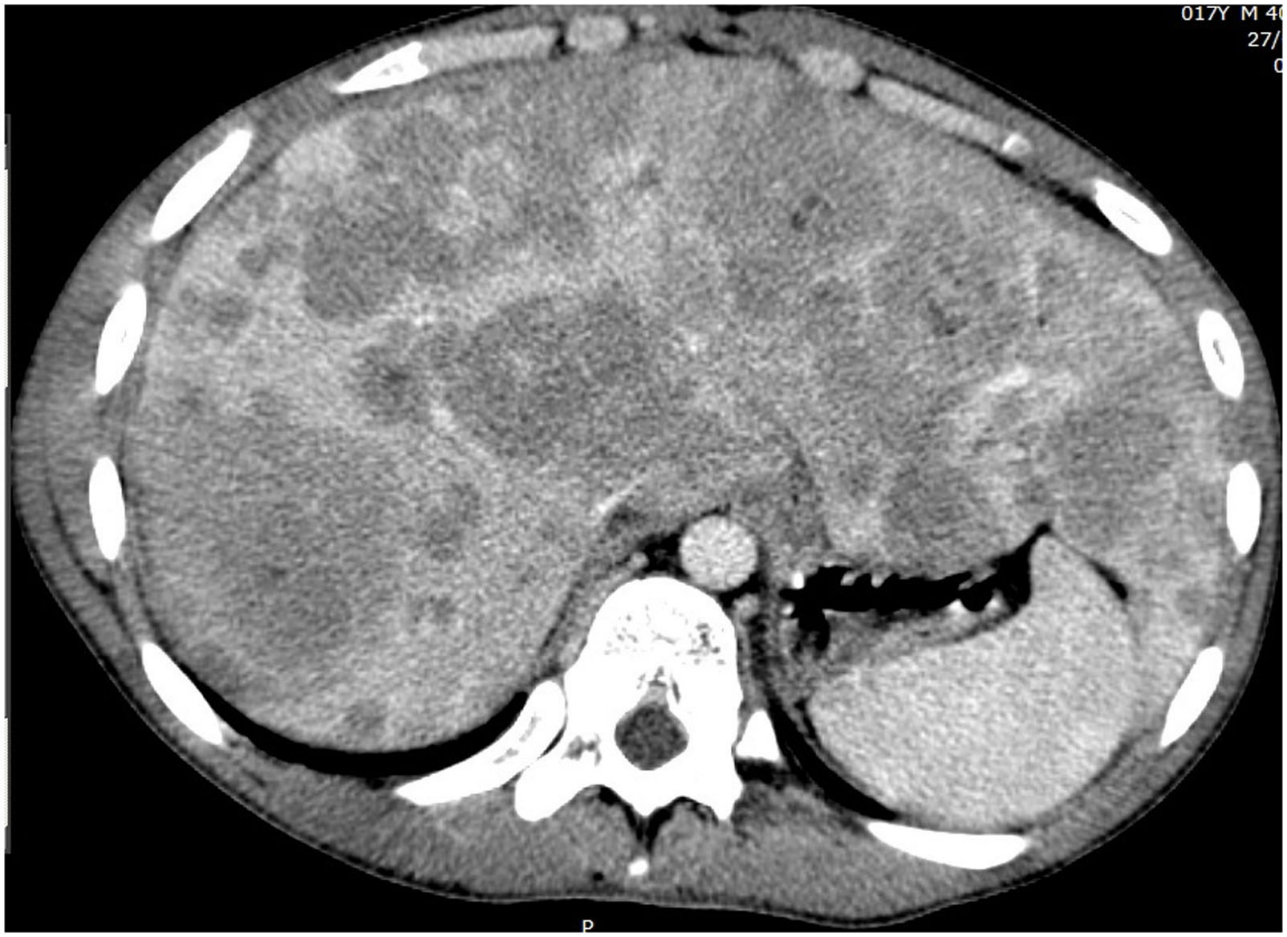
**Abdominal CT with the multiple liver metastasis**.

**Figure 2:**
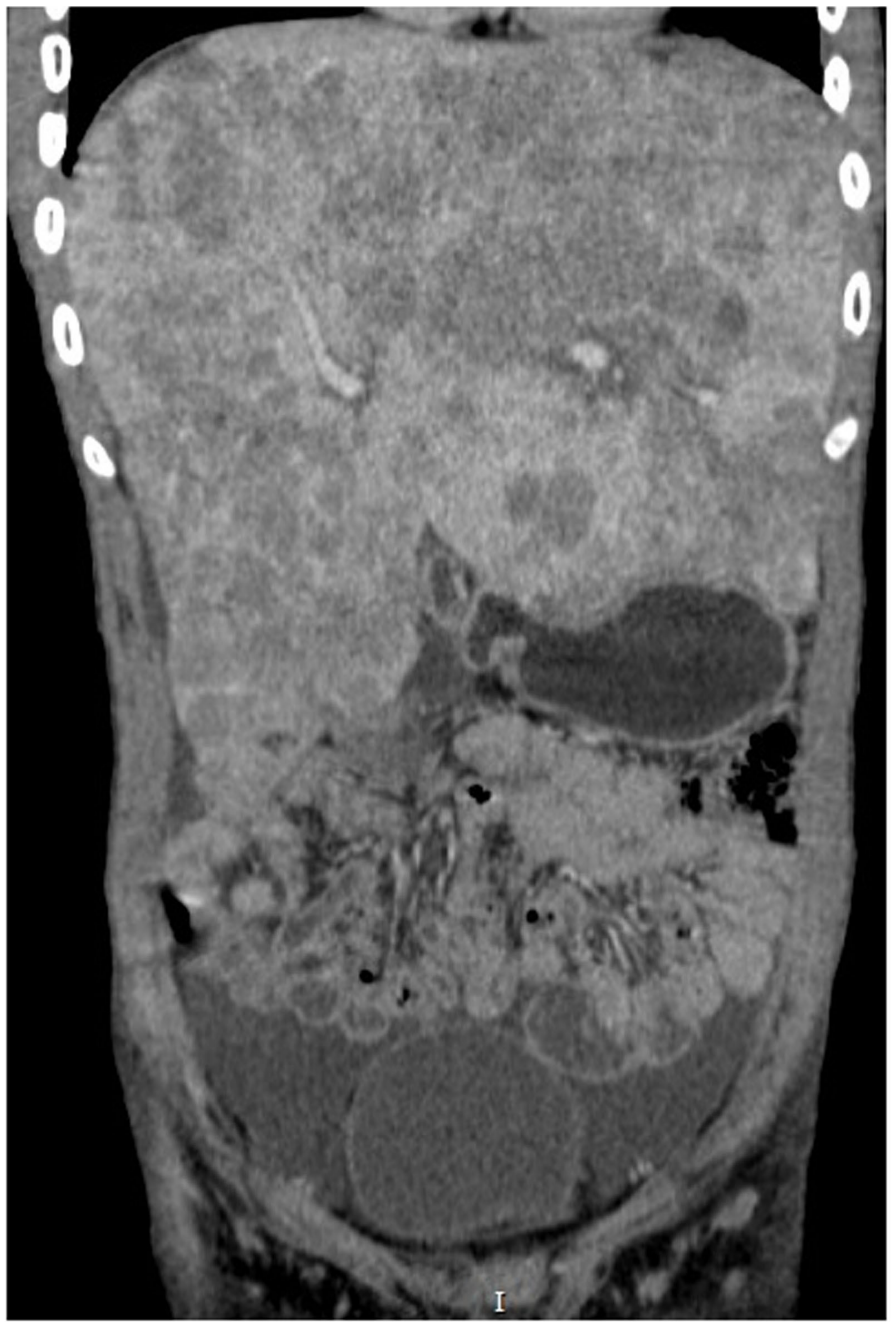
**Abdominal CT in coronal cut showing the massive hepatomegaly**.

Abdominal CT scan (Figures 1 and 2) showed slight peripheral impregnation by contrast in the innumerous hypodense lesions, in addition to lymph node enlargement in the upper abdomen and pelvis. Low to moderate amount of free fluid was seen in the abdominal cavity. Besides all this complementary investigation, the patient initiate to complain about right eye pain, the blinded eye traumatized 4 months ago.

**Figure 3:**
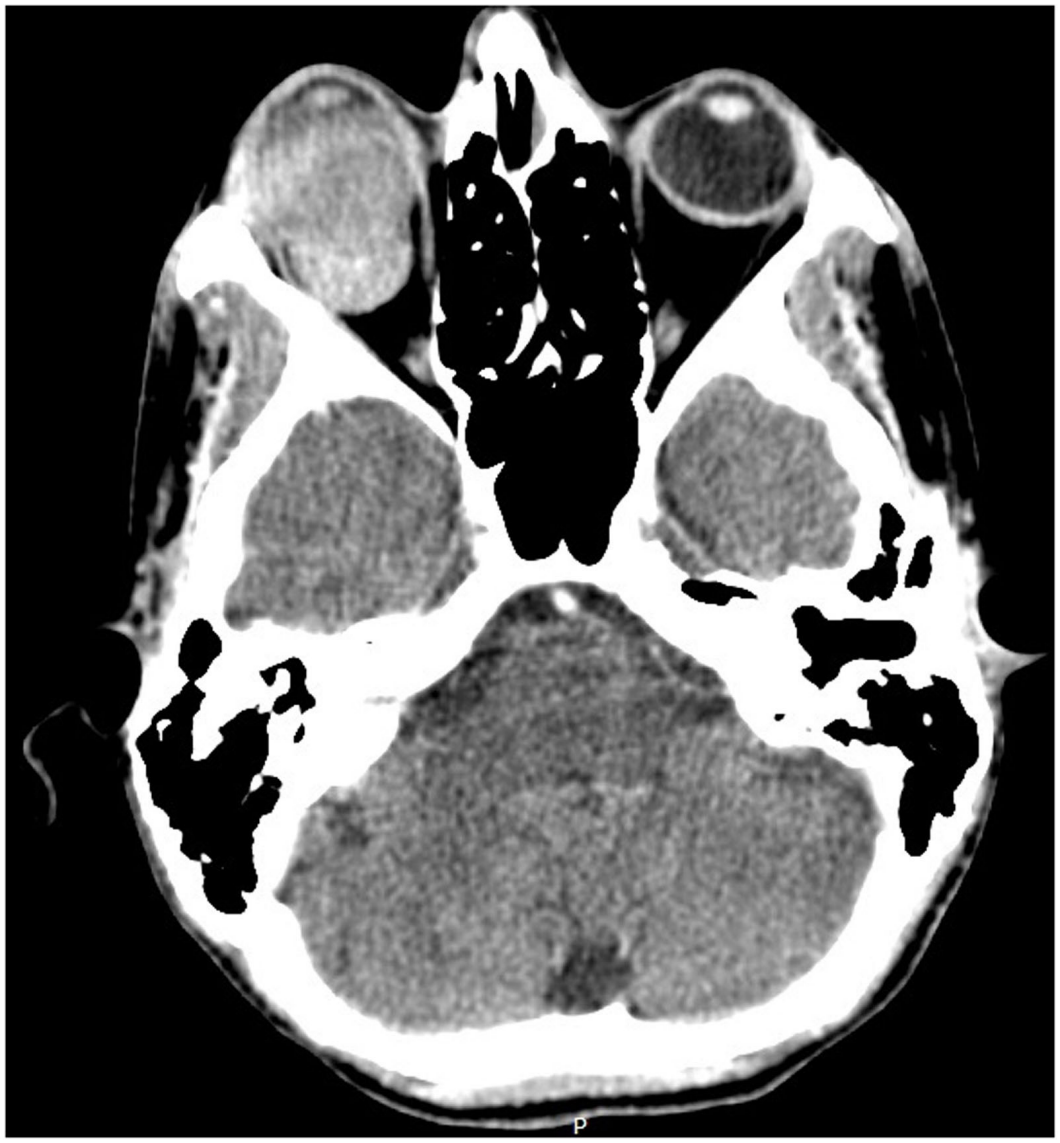
**Orbital CT with the expansive lesion in the right eye**.

The funduscopic examination of the right eye revealed a choroidal detachment and was not possible to define the etiology, probably traumatic. Orbit tomography (Figure 3) demonstrated right exophthalmos associated with a spontaneously expansive lesion without significant contrast enhancement, with the center located in the posterior chamber of the right eye, measuring approximately 3.0 × 2.6 cm (anteroposterior × transverse).

During this investigation, the hepatic biopsy showed poorly differentiated neoplasm of polygonal cells of intermediate size, anisocariosis, and oval nucleus exhibiting evident nucleolus. Rare neoplastic cells appear to exhibit dark-to-black cytoplasmic brown pigment. Immunohistochemistry assay revealed melanoma-compatible pattern: [PS100 (anti-humanS-100):positive/HMB45 (cloneHMB45):positive focal/Melan-A (cloneA103) positive].

During the entire hospitalization, patient remained with the complaint of only nausea and vomiting. Fungal lesions initially presented throughout the dorsum improved after topical treatment. Despite antiemetic measures, patient showed worsening complaints of nausea and vomiting, with abdominal pain initiating in this context. Due to the symptomatic picture, low effectiveness in this scenario and unavailability for the promptly start of immunotherapy in our public health system, we decided to initiate carboplatin and paclitaxel chemotherapy adjusted for renal and hepatic function. However, the patient developed fulminant hepatic failure in less than 24 h after the cytotoxic administration, presenting with encephalopathy, abrupt coagulation disorders, increased liver enzymes to >1,000 IU/L. Palliative analgesia was initiated, and the patient died within 48 h.

## Background

### Epidemiology

Uveal melanoma is a relatively rare malignancy, accounting for approximately 5.1 million cases/year (1). The uveal tract is composed of iris, ciliary body, and choroid, with uveal melanoma developing in any of these parts, after mutations in the melanocytes of this layer. Approximately 95% of ocular melanomas are found on the uvea and the remainder on the conjunctiva. This tumor variant has significant differences in relation to cutaneous melanoma, in terms of pathophysiology, prognosis, and epidemiology. The mean age at diagnosis is 60 years (1, 2), being rare among young patients. According to the data available in the literature, 0.5–1.3% of patients with uveal melanoma are younger than 20 years (3–5), as is the case in question. When compared with elderly patients, the younger ones are still less likely to present with metastatic disease (1). Uveal melanoma mainly affects the white population, mainly of high latitudes (6), and the proportion of whites:blacks reaches up to 196:1 in some analysis (1). Individual characteristics such as inability to tan, light skin, blue eyes, and sunburn represent risk factors associated with a higher incidence of uveal melanoma (7). Choroidal nevi are a present alteration in up to 3% of people older than 30 years (8) and are a risk factor well related to the appearance of uveal melanomas (9).

### Pathophysiology

The pathogenesis of this variant of melanoma differs from the cutaneous form in some aspects, being much less studied until now. It is known that uveal melanoma does not have a high frequency of *BRAF* mutations. By contrast, mutations in *GNAQ* or *GNA11* (G protein subunit coders) are expressed in about 80% of the cases, being responsible by the cascade of *MAPK* pathway activation (10–12).

Genetic determinant prognostic changes include mutations of *BRCA1*-associated protein—present in 84% of metastatic cases (13). A good prognosis has been associated with mutations in the *SF3B1* gene, present in 18.6% of primary uveal melanomas (14). Among clinical risk factors, the studies indicated skin phenotype, predisposition to sunburn, and clear eyes (7). Chronic ultraviolet exposure associated with activities such as welding increases the risk of uveal melanoma development (15). Choroidal nevi also are correlated with a risk of malignant transformation according to ophthalmologic measuring parameters.

## Discussion

### Diagnosis

Intraocular tumors usually presents with visual complaints. Patients seek the ophthalmologist or general practitioner referring blurred vision, photopsia, visual scotomas, and even amaurosis. The diagnosis is based on the funduscopic examination performed by an experienced ophthalmologist, the most accurate factor for diagnosis of this condition. Biopsy is generally not necessary, and ocular globe ultrasonography is the most important complementary modality, usually eliminating the need for an invasive diagnosis. Pathologic analysis is reserved for diagnostic dilemmas, where it can drastically change the case’s subsequent management (16). In differential diagnosis, primary tumors of other sites with potential to send metastasis to the uvea, such as lung and breast, should be considered (17, 18). Regarding staging, TNM system is classically employed (19). The risk of developing metastasis increases with the size of the primary tumor at diagnosis, as well as the probability of death within 10 years (20).

### Treatment of Initial Disease

Radiotherapy achieves local tumor control in most cases, when compared to survival-related outcomes achieved by enucleation (21–24). This can be performed by brachytherapy or external field radiation. In the light of current evidence, enucleation therapy is now reserved for patients with extensive tumors and/or local complications. There also appears to be no benefit in preenucleation radiotherapy (25). Other techniques used in the management of uveal melanoma include localized resection, transpupillary thermotherapy, photodynamic therapy, and photocoagulation.

Although local treatment is effective in preventing local recurrence in approximately 95% of cases, approximately 50% of patients develop distant metastasis within 5 years after enucleation (26). When treated with ocular globe irradiation, the subsequent onset of metastasis reaches 23–50%, depending on prognostic factors involved (27, 28), i.e., increased tumor diameter, extrascleral extension, ciliary body involvement, advanced age, iris color, tumor pigmentation, symptomatic disease, and chromosomal monosomy 3 (28–39). Adjuvant therapy has no defined role, and some of the scanty evidence even brought negative results with adjuvancy, such as the study by Lane et al. (40).

Uveal melanoma spreads essentially by hematogenic path, due to the absence of lymphatic drainage in the uveal tract. Unlike cutaneous melanoma, the most common site of systemic dissemination is the liver, present in up to 90% of cases, as exemplified by our case. Genetic inheritance, histological grade, primary lesion size, and ciliary body involvement are some of the factors that increase the risk for systemic disease (28, 41). In the presence of such factors, and even in the absence of these factors, some experts advocate long-term follow-up because of the possibility of late metastasis arising in the disease (42). The prognosis in patients detected with metastatic disease becomes poorly, varying between 2 and 12 months for median survival in the literature (43, 44). Treatment of metastatic disease remained disappointing till recent years. Up to 80% of patients are dead after 1 year of diagnosis of metastasis and 90% in 2 years, with an average survival time of less than 6 months (45).

The role of systemic chemotherapy shows non-convincing benefits for cases of uveal melanoma. Therefore, the early detection of late metastasis does not have an impact on the survival of these patients due to the scarcity of treatments for the disseminated disease (46).

### Treatment of Metastatic Disease

There is no therapeutic consensus for those patients who develop metastasis. Despite the use for cutaneous melanoma, the results of the most varied chemotherapy schemes and drugs were not shown to be encouraging with drugs such as dacarbazine, cisplatin, temozolomide, treosulfan, and others in the most varied combinations.

#### Anti-CTLA4 Antibody

Ipilimumab was evaluated by the Spanish Melanoma Group in 32 patients with treatment-naive metastatic disease. After 5.5 months, of the 13 patients available for evaluation, only 1 had partial response and 6 had stable disease. Mean overall survival in this study was 9.8 months (47). Another phase II study involved 45 previously treated patients and 8 virgin patients with metastatic uveal melanoma, performed by Zimmer and colleagues (48). Of these, six patients maintained stable disease, while none experienced any response at any level. Overall median survival of the study was 6.8 months, and median time to progression was 2.8 months. A study carried out by Maio et al. with 82 patients achieved PFS of 3.6 months, but with overall survival of 6.0 months (49). A study published in 2013 by Luke et al. (50) was able to demonstrate sustained response rates with the use of ipilimumab in this setting, at the expense of higher rates of long-term manageable adverse events. The response rate at 12 weeks was only 2.6%, reaching 46% when we added the stable disease tax. At 23 weeks, this response decreased to 28.2% of cases. The median survival in this study was 9.6 months (95% CI 6.3–13.4), summing better results for this scenario.

More recently, tremelimumab (another fully humanized anti-CTLA-4 antibody) had a study with its evaluation of efficacy terminated early due to futility after presenting a 2.9-month PFS (corresponding to 9.1% PFS rate in 6 months) (51).

#### Anti-PD-1 and Anti-PD-L1 Therapy

Pembrolizumab, an anti-PD1 antibody, was evaluated in a small number of patients (10 in total) in the study by Kottschade et al. (52). In this series of cases, the median PFS was 4.5 months, reaching one complete response case, two cases of partial response, and one patient with stable disease among eight evaluated at the end of follow-up. The most robust results involving modern immunotherapy in uveal melanoma come from the collection by Algazi et al. (53), involving 48 patients exposed to previous chemotherapy and 35 patients already exposed to ipilimumab who received immunotherapy with pembrolizumab (38 patients), nivolumab (16 patients), and atezolizumab (2 patients). Objective tumor response was achieved in only two patients (overall response rate 3.6%) and stable disease in 5 patients (9%). In this study, overall survival was 7.6 months (95% CI 0.7–14.6 months) and PFS was 2.6 months (95% CI 2.4–2.8 months).

#### Molecular Target Agents

Although uveal melanoma does not express direct changes in the *BRAF* gene (rendering its indication of vemurafenib and dabrafenib unfeasible), it has mutations in the *GNAQ* or *GNA11* (G protein subunit encoding) genes within 80% of cases. This lead to the activation of the cascade of *MAP* kinases also resulting in proliferation and tumor growth. A phase II trial conducted by Carvajal et al. (54) randomized 101 patients to compare selumetinib (*MEK* inhibitor) vs temozolomide with dacarbazine upfront. The progression-free survival period was substantially increased in the selumetinib arm (15.9 vs 7 weeks: HR 0.46, 95% CI 0.3–0.71). The objective response rate was also favorable (14 vs 0%), and although not statistically significant, median overall survival was increased (11.8 vs 9.1 months; HR 0.66, 95% CI 0.41–1.06, *p* = 0.09). However, the subsequent phase III study, SUMIT (55), which sought to access the efficacy of selumetinib + dacarbazine vs placebo + dacarbazine in the first-line treatment of metastatic uveal melanoma, failed to reach the primary endpoint, i.e., PFS (2.8 vs 1.8 months; HR 0.78, 95% CI 0.48–1.27, *p* = 0.32) in an evaluation performed by the independent review center of the study.

Other studies comparing and associating inhibition of the *MAPK* pathway with other chemotherapeutic agents are underway. Another inhibitor of the *MEK* family, trametinib, was under evaluation in a phase I study (56) but demonstrated limited clinical efficacy against previously treated metastatic uveal melanomas (PFS of 1.8 months and global response rates of approximately 0%). Another possibility that arises is the association of *MEK* and *AKT* double inhibition (whose phosphorylation is observed in >50% of uveal melanomas). This strategy is currently being evaluated in more than one study. *GNAQ* and *GNA11* lead mutations generate an upregulation of *MET* factor (related to the appearance of liver metastasis by uveal melanoma). A subgroup analysis of 23 patients treated with cabozantinib (non-selective dual inhibitor of *MET* and *VEGF*) as part of a phase II trial (57) revealed an overall survival of 12.6 months and a PFS of 4.8 months. This led to the development of a specific phase II trial for the uveal subtype of melanoma.

## Concluding Remarks

We report this case because of the rarity of metastatic uveal melanoma presentation in a 17-year-old patient. The unfortunate fact of this advanced and very rapid presentation could have as a contributor the missed diagnosis of the primary tumor due to the absence of visual symptoms caused by a previous local trauma. Most likely, this lesion was already developing at the time of the trauma, not being detected at the time of the medical evaluation or being confused with trauma artifacts, most likely with hepatic implants already in development 4 months earlier. The high-speed unfavorable evolution of this case was determined by an advanced liver involvement.

We emphasize the importance of ocular examination and the consideration of uveal melanoma in the differential diagnosis of cases with metastatic neoplasia without defined primary focus, even at early ages, and especially when presenting isolated liver metastasis. This report serves as an alert for pediatricians and other oncologists to the fact that there is a very rare but possible presence of uveal melanoma in the youth.

## Ethics Statement

The patient and his legal representatives signed a consensus agreement authorizing the medical oncologist to use their medical records and images in the case report, preserving the patient’s identity.

## Author Contributions

PB: physician who assisted the patient during his hospitalization and was responsible for compiling data. SA: medical advisor.

## Conflict of Interest Statement

The authors declare that the research was conducted in the absence of any commercial or financial relationships that could be construed as a potential conflict of interest. The reviewer, MR, and handling editor declared their shared affiliation, and the handling editor states that the process nevertheless met the standards of a fair and objective review.
